# An Estimate of the Onset of Beadless Character of Electrospun Nanofibers Using Rheological Characterization

**DOI:** 10.3390/polym13020265

**Published:** 2021-01-14

**Authors:** Petra Peer, Jana Zelenkova, Petr Filip, Lenka Lovecka

**Affiliations:** 1 Institute of Hydrodynamics, Czech Academy of Sciences, 166 12 Prague, Czech Republic; zelenkova@ih.cas.cz (J.Z.); filip@ih.cas.cz (P.F.); 2 Centre of Polymer Systems, University Institute, Tomas Bata University in Zlin, 760 01 Zlin, Czech Republic; lovecka@utb.cz

**Keywords:** electrospinning, PVF-*co*-HFP, PVB, PEO, viscoelasticity, specific viscosity

## Abstract

Electrospinning represents the very effective process of producing nanofibrous mats. This process is influenced by a number of mutually and strongly interlaced entry parameters (characteristics of polymer, solvent, process parameters) and their participation in the resulting nanofiber quality. The appearance of nanofibers is a result of the necessary primary experimental parameter setting within an acceptable range. However, finer analysis of nanofiber quality depends on the proper choice of these individual factors. The aim of this contribution is to evaluate one of the key factors—polymer concentration—with respect to the presence or absence of bead formation. This passage can be approximated by rheological oscillatory measurements when a sudden decrease in phase angle indicates this change. It replaces otherwise time- and cost-consuming trial-and-error experiments. This approach was tested using three different materials: solutions of poly(vinylidene fluoride-*co*-hexafluoropropylene), poly(vinyl butyral), and poly(ethylene oxide).

## 1. Introduction

In past decades the application of nanofibrous materials has shifted from classical filters to other uses such as protective clothing, antibacterial wound dressing, and tissue engineering, to name a few. In some applications the appearance of singularities along the nanofibers, so called beads characterized by an abrupt increase in nanofiber cross-section followed by a sudden decrease, is required as it substantially improves adhesion [1,2]. However, the majority of production aims for a smooth constant profile of nanofibers along their length.

The electrospinning process [3,4,5,6,7,8] represents one way to produce nanofibers in a relatively cheap manner. This process is based on applying a high voltage (in orders of ten kV) to polymer solutions or melts when ejected nanofibers are deposited on a grounded collector. Quality of the resulting nanofibers is subject to various parameters that can be classified to four basic groups: polymer characteristics, solvent characteristics, solution characteristics, and process parameters. 

This concerns, for instance, an appearance of beads along the individual nanofibers, which is mostly an unwanted phenomenon. However, in some cases an appearance of beads supports an application of nanofibers, as in the case of improving adhesion [1,2]. Bead formation strongly depends on various factors such as voltage [9], tip-to-collector distance [9], and additives [10,11]. A choice of solvents also contributes to this phenomenon [10,12]. This influence can be quantified through the so-called Hansen solubility parameters [13], indicating a mutual relation between the polymer and the thermodynamic quality of solvent (good, poor). Appearance or suppression (elimination) of bead formation is also influenced by used techniques, for instance, by hot stretching [14].

One of the crucial parameters is represented by the polymer concentration. Its value contributes to distinguishing the visual properties of the final product to three distinct cases: electrospraying (discrete drops), continuous nanofibers, and the creation of spots produced by spinning due to incomplete solvent evaporation. De Gennes [15] set four consecutive regions of polymer solutions differing in concentration: dilute, semidilute unentangled, semidilute entangled, and concentrated. The first two regions are separated by the so-called overlap concentration c*, the semidilute regions by the so-called entanglement concentration ce. For individual materials the multiplicative coefficients k of entanglement concentration ce (where a semidilute entanglement region starts) are determined in such a way that for concentrations lower than k.ce the electrospun nanofibers exhibit singularities (appearance of beads along the nanofibers), and for concentration higher than k.ce the beads are suppressed and the nanofibers exhibit a relatively smooth surface. Most multiplicative coefficients exceed a value of 1 [16], Table 1, as e.g., k = 8 for nylon 6 [17], k = 1.37 for polyimide [18], but values lower than 1 for some materials are also reported as, e.g., k = 0.8÷1 for poly(ethylene terephthalate) [19]. A value of the coefficient k varies dependent on the polymer solutions used, and its determination is based on the experiments carried out for a series of concentrations.

The question is whether or not this method of evaluating an onset of concentrations for which beadless nanofibers are produced can be simplified. To this aim we chose three frequently used materials:

(a) copolymer of poly(vinylidene fluoride) and hexafluoropropylene (PVDF-*co*-HFP) dissolved in *N*,*N*’-dimethylformamide (DMF),
(b) poly(ethylene oxide) (PEO) dissolved in water, and
(c) poly(vinyl butyral) (PVB) dissolved in ethanol.

ad (a)

Thermoplastic fluoropolymer poly(vinylidene fluoride) (PVDF) is widely used in various applications due to its unique properties compared to other polymers. It is resistant to aggressive conditions due to its high chemical, thermal, and UV stability. Further, PVDF exhibits very good mechanical strength, high hydrophobicity, thermal stability, and low density (only 1.78 g/cm^3^ in comparison with polytetrafluoroethylene (PTFE)—2.2 g/cm^3^). Even more attractive is a copolymer of PVDF and hexafluoropropylene (PVDF-*co*-HFP), which is characterized by higher solubility, lower crystallinity, better mechanical strength, and higher hydrophobicity. In addition, this copolymer exhibits the highest dielectric constant and electroactive response, including piezoelectric, pyroelectric, and ferroelectric effects. All these properties predetermine PVDF-*co*-HFP as a very perspective material in membrane production. PVDF-*co*-HFP membranes are used especially in two spheres: membrane distillation (e.g., in the desalination process, where the membranes gradually replace reverse osmosis) [16] and in energy storage devices in the form of separators in lithium-ion batteries. Novel trends also find application in biomedicine, e.g., encapsulation of bacteria in core-shell structures [20] or in endothelialization [21].

ad (b)

Poly(ethylene oxide) is among the most easily spinnable materials. Moreover, its excellent biodegradability, biocompatibility, and non-toxicity predetermine electrospun PEO nanofibrous mats to applications in biomedicine and the food industry. The relatively small participation of PEO (up to 2%) also enables spinnability of otherwise unspinnable or very problematically spinnable materials such as chitin or chitosan [22,23,24,25]), keratin [26,27], silk [28,29], and other materials.

ad (c)

Poly(vinyl butyral) exhibits features that are similar to those introduced above for PEO. PVB is an odorless, nontoxic polymer, fully biocompatible, and characterized by flexibility and good adhesion to various substrates. It predominantly serves as an interlayer material [30,31] in car windscreens (2/3 of its production is used in the automotive industry). Due to its properties PVB is often used in food packaging, and as excellently spinnable material [32], is also added to improve the spinnability of other materials [33].

Based on oscillatory rheological measurements, the aim of this contribution is to show a close correlation between the onset of a starting concentration for which a presence of beadless nanofibers dominates, and a location of phase angle decrease, i.e., a sudden decrease from the constant course or only a moderate decrease in the curve phase angle vs. the concentration of polymer solution. 

## 2. Materials and Methods

### 2.1. Material

Kynar Flex^®^ 2801 (copolymer poly(vinylidene fluoride)-*co*-hexafluoropropylene), datasheet [34], was purchased from Arkema (Colombes, France), and *N*,*N*′-dimethylformamide (DMF) (p.a.) was purchased from P-LAB, a.s. (Prague, Czech Republic). 

Poly(vinyl butyral) Mowital B 75H, datasheet [35], was purchased from Kuraray Specialities Europe, and ethanol (quality of p.a.) from Penta (Prague, Czech Republic). The structure of Mowital B 75H (the suffix H indicates the degree of acetalization) is composed of vinyl butyral, vinyl alcohol, and vinyl acetate, in this case 75–81%, 18–21%, and 0–4%, respectively. 

Poly(ethylene oxide), datasheet [36], was purchased from Sigma Aldrich (St. Louis, MO, USA) and dissolved in distilled water. 

All chemicals were used as obtained without further refinement.

### 2.2. Preparation of Electrospinning Solution

Polymer materials were dissolved in corresponding solvents (Table 1) using a magnetic stirrer MR Hei-Tec (Heidolph, Schwabach, Germany) with the help of a Teflon-coated magnetic cross under these conditions: mixing rate was 250 rpm, temperature 25 °C, and time of mixing was 48 h (24 h in the case of Kynar Flex^®^ 2801). 

The concentrations of all prepared solutions and molecular weights of used polymers are summarized in Table 1. Relatively wide ranges of concentrations were chosen for each polymer. These ranges cover most possibilities of nanofiber quality, starting from relatively low concentrations providing either no or unacceptable nanofibers. Low concentrations resulted in low viscosity of prepared polymer solutions where the individual macromolecular chains did not influence their neighbors. Such polymer solutions did not indicate any elasticity as verified by the rheological oscillatory measurements. On the other hand, high concentration implied a significant increase in viscosity, which can result in the suppression of Taylor’s cones and, hence, in emanation of viscoelastic jets. The sufficiently wide and dense concentration range ensured that the full palette of nanofibers was at our disposal; moreover, it ensured an acceptable approximation of a location where a phase angle started to decrease.

### 2.3. Process of Electrospinning

**Figure 1:**
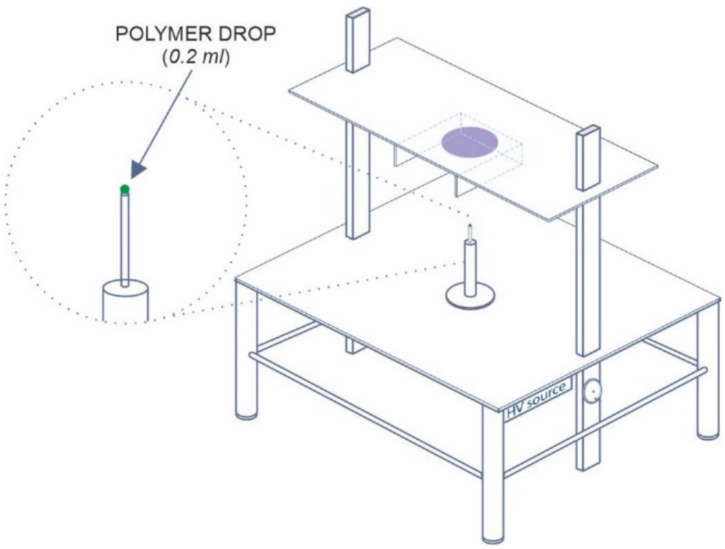
Schematic sketch of the needleless electrospinning device.

The nanofibers were spun using our laboratory needleless device (see Figure 1) equipped with a high-voltage power supply SL70PN150 (Spellman, Hauppauge, NY, USA), a carbon steel stick (10 mm in diameter) with a semispherical hole for depositing of 0.2 mL of polymer solution, and a motionless flat metal collector, for details see [37]. 

The three setup orientations for electrospinning are vertical top-down, vertical bottom-up, and horizontal. These orientations will change the mutual configurations between the gravitational and electric fields. Whilst it appears that the intensity of the gravitational field is negligible in comparison to the electric, some studies indicate that gravity should be taken into account. Yarin et al. [38] showed larger values of Taylor cones for bottom-up configuration in comparison with top-down arrangements. Yu et al. [39] showed that the opposition of gravitational and electrostatic forces (bottom-up geometry) contributed to suppressing Rayleigh and whipping instabilities. The topic of gravity is discussed in further detail within the review by Suresh et al. [40].

The individual materials were electrospun under the following conditions.

PVDF-*co*-HFP/DMF—The electrospinning process was carried out at a voltage of 18 kV with the fixed tip-to-collector distance of 100 mm at ambient conditions of 23 ± 1 °C and a relative humidity of 34 ± 1%.

PVB/ethanol—The following process parameters were fixed: the tip-to-collector distance was 100 mm, ambient temperature 21 ± 1 °C, and relative humidity 40 ± 1%. The voltage was fixed to 20 kV ensuring generation of fibrous mats without presence of blobs (sufficient rate of solvent evaporation).

PEO/water—The electrospinning process was carried out at a voltage of 12–25 kV with the fixed tip-to-collector distance of 200 mm at ambient temperature of 22 ± 1 °C and a relative humidity of 39 ± 2%.

### 2.4. Rheological Measurements

A rotational rheometer Physica MCR 501 (Anton Paar, Graz, Austria) equipped with the concentric cylinder geometry (the inner and outer diameters were 26.6 and 28.9 mm, respectively) was used both for oscillatory measurements (frequency sweep within 0.1–100 Hz at strain 1%) providing elastic *G*’ and viscous *G*’’ moduli, and for shear viscosity measurements (a range 0.01–300 s^−1^). The value of shear rate $\dot{\gamma }$ = 0.12 s^−1^ belonging to a linear viscoelastic region was chosen for measurement of shear viscosity of polymer solutions with different polymer concentrations, and consequently applied to a determination of specific viscosity. The phase angle *δ* (tan *δ* = *G*’’/*G*’) was determined at a frequency of 10 Hz. The temperature was set to 25 °C. Each measurement was carried out at least three times and the individual runs were more or less identical.

The measured shear viscosities of all three solvents were compared with responsible literature (referred to in Section 3), and the results proved a very good correspondence. The viscosities for polymer solutions cannot be compared with data presented in the literature as molecular weights of polymeric materials differ from batch to batch. Consequently, as rheological characteristics strongly depend on molecular weight, it is necessary to repeat their determination for every batch. This concerns not only shear viscosity but especially the viscoelastic behavior of polymer solutions. Hence, courses of a curve phase angle vs. concentration can differ with new material.

### 2.5. Characterization of Nanofibrous Mats

A high-resolution scanning electron microscope Vega 3 (Tescan, Czech Republic) was used for characterization of nanofibrous mats. Prior to imaging, the samples were sputtered by a conductive coating layer using a sputter Quorum Q150R (Quorum Technologies Ltd., Laughton, UK). 

## 3. Results

In the following, interlacing of rheological characteristics of polymer solutions used in the process of electrospinning with the morphological characterization of electrospun nanofibers are presented with an emphasis to create nanofibers with a good (bead-free) quality. 

### 3.1. PVDF-co-HFP Nanofibres

**Figure 2:**
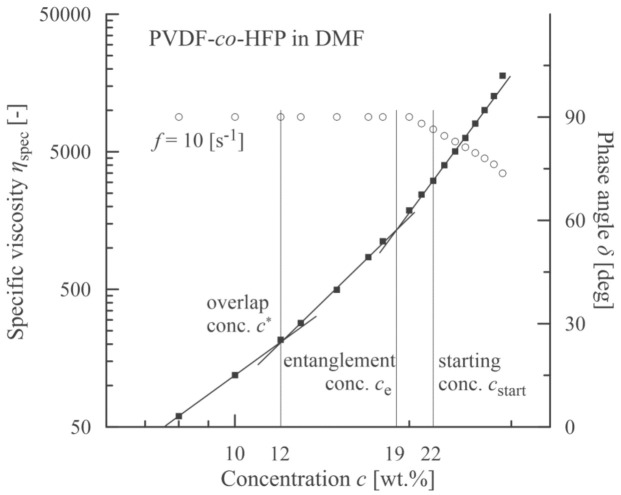
Correlation between an onset of viscoelasticity (a decrease in phase angle, open circles) and a starting concentration corresponding to an onset of beadless PVDF-*co*-HFP nanofibers.

Based on measurement of shear viscosities for individual concentrations of PVDF-*co*-HFP, the specific viscosities were calculated using the relation *η*_sp_ = (*η*0–*η*_s_)/*η*_s_, where *η*0 is the zero-shear-rate viscosity (measured at $\dot{\gamma }$ = 0.12 s^−1^) and *η*_s_ is the solvent (DMF) viscosity (=0.807 mPa·s). This measured value corresponds to the values presented in the literature [41,42]. Consequently, the first three concentration regions according to de Gennes [15] were determined separated by the overlap concentration c* (≈12 wt.%) and entanglement concentration *c*_e_ (≈19 wt.%). These two concentrations correspond to the intersection points of linear segments optimized with respect to the experimental points, see Figure 2.

Based on elastic (storage) *G*’ and viscous (loss) *G*” moduli, a course of the phase angle *δ* (tan*δ* = *G*”/*G*’) is depicted in Figure 2. A value of concentration for which the phase angle starts to decrease (slightly more than 20 wt.%) corresponds to the formation of network structures among the polymer chains and exceeds a location of the entanglement concentration (≈19 wt.%).

**Figure 3:**
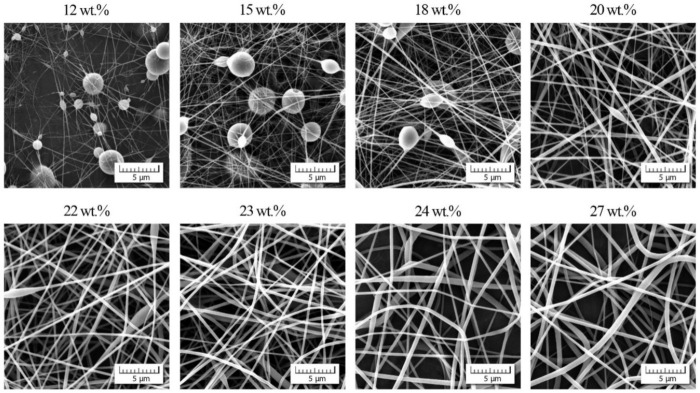
Morphology of individual nanofibers in dependence on PVDF-*co*-HFP concentration.

The morphology of PVDF-*co*-HFP nanofibers was correlated with the rheological characteristics of measured solutions. Figure 3 documents that up to the overlap concentration (c* ≈ 12 wt.%) no nanofibers were formed, and as a result, only a disordered set of blobs were received. Starting with the overlap concentration the primary forms of nanofibers were indicated mixed with the blobs, and finally a passage from bead to bead-free nanofibers was apparent around the entanglement concentration. It seems that an adequate quality of nanofibers was achieved for *c*_start_ = 22 wt.% and higher. This value closely corresponded to a concentration for which the phase angle started to decrease as elasticity began to manifest.

### 3.2. PVB Nanofibres

**Figure 4:**
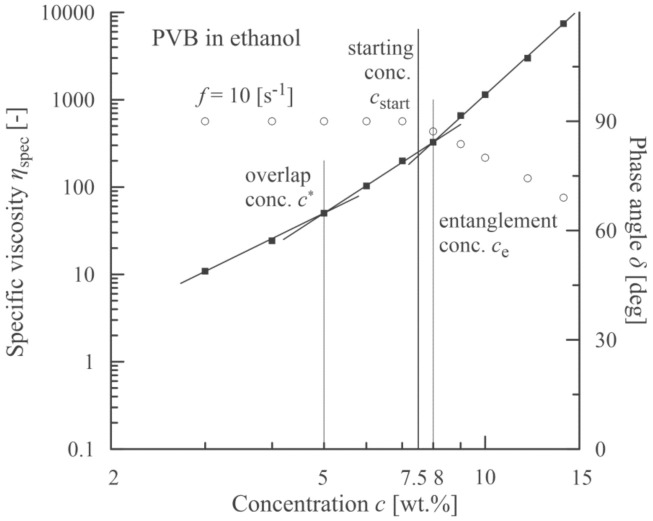
Correlation between an onset of viscoelasticity (a decrease in phase angle, open circles) and a starting concentration corresponding to an onset of beadless PVB nanofibers.

**Figure 5:**
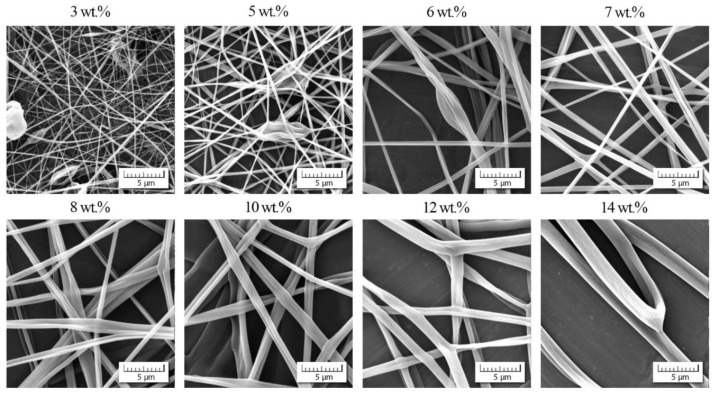
Morphology of individual nanofibers in dependence on PVB concentration.

The analogous experimental approach was also carried out with PVB dissolved in ethanol (a measured value *η*_s_ = 1.087 mPa·s corresponds to data in literature [43,44]). The results are introduced in Figure 4 and Figure 5. In this case the starting concentration was slightly lower than the entanglement concentration, which was similar to poly(ethylene terephthalate) [19].

### 3.3. PEO Nanofibres

**Figure 6:**
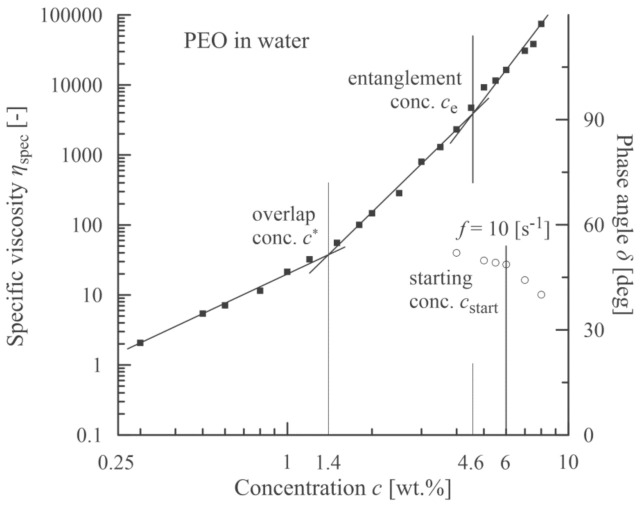
Correlation between a sudden decrease in phase angle (open circles) and a starting concentration corresponding to an onset of beadless PEO nanofibers.

**Figure 7:**
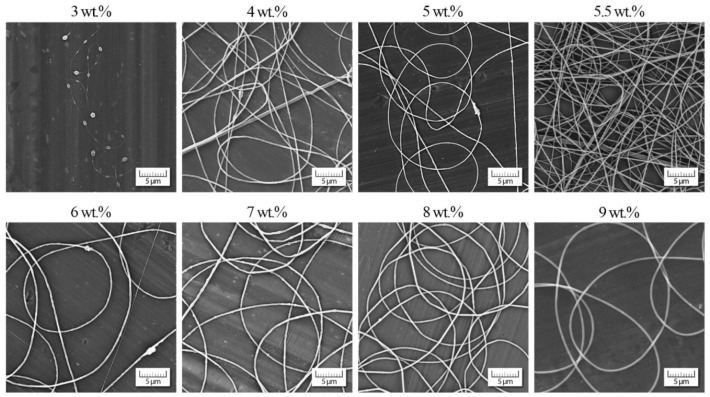
Morphology of individual nanofibers in dependence on PEO concentration.

The analogous experimental approach was also carried out with PEO dissolved in distilled water (*η*_s_ = 0. 890 mPa·s according to [45]). The results are summarized in Figure 6 and Figure 7. In this case (*M*_w_ = 637,500 g/mol) the polymer solutions also exhibited viscoelastic behavior for lower concentrations due to higher molecular weight. At first, the decrease in the relation phase angle vs. concentration was rather moderate. Then, at the concentration of approximately 6% a sudden drop was apparent. Hence, this value (*c*_start_) indicates the region from which the nanofibers have a dominantly bead-free characteristic.

### 3.4. Summary of the Individual Results

Table 2 summarizes the results for all three materials. The differences between the individual polymers were also subject to topology of macromolecular chains and their possible entanglement.

The determination of the starting concentration for the PEO solution is not so strict for the remaining two materials as PEO solutions also exhibit viscoelastic behavior for lower concentrations due to their relatively high molecular weight. A more pronounced decrease in the phase angle starts from an already mildly decreasing curve phase angle vs. concentration which contrasts to the PVDF-*co*-HFP and PVB solutions, for which the phase angle attains a constant value (90°) for lower concentrations. This also results in the relatively gradual passage of PEO nanofibers from beaded to beadless character. 

## 4. Discussion

Possible electro-spinnability of polymeric materials and quality of the resulting nanofibers are subject to a number of entry parameters, which can be roughly distributed into four categories: polymer characteristics (molecular weight, viscosity, etc.), solvent characteristics (rheological parameters, etc.), polymer solution characteristics (concentration, Hansen solubility parameters, etc.), and process characteristics (e.g., voltage, tip-to collector distance, temperature, humidity).

**Figure 8:**
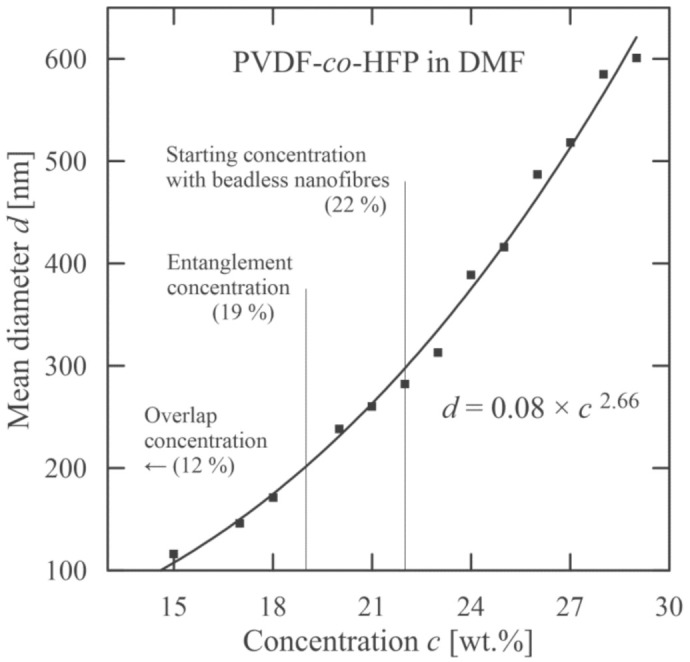
Dependence of mean nanofiber diameter on PVDF-*co*-HFP concentration.

These characteristics are mutually interlaced. This means that it is not possible to alter only one, as for instance, viscosity strongly depends on molecular weight and concentration. This is also connected with an increase in nanofiber diameter. The algebraic expression relating a mean nanofiber diameter with variable molecular weight and concentration for PVB is introduced in [46], and for PEO in [47]. An increase in mean diameter with concentration for used copolymer PVDF-*co*-HFP (fixed molecular weight) is illustrated in Figure 8.

Above, it is implied that a specific polymeric material is only necessary to access experimentally in order to derive a suitable setting of entry parameters. However, for a given relatively acceptable set of entry parameters, there is a question as to whether a local tuning of one chosen parameter cannot be based on purely instrumental analysis (rheological measurements), and thus eliminate time-consuming and expensive trial-and-error experimental methods. The explicit expressions that relate a distinct parameter to adjustable parameters—providing the first approximation—can serve to simplify and assist in the orientation of the whole problem. For example: to determine the nanofibers’ mean diameter, which is dependent on a variable molecular weight and concentration, as indicated above, and similarly, to determine the starting concentration cstart which is dependent on molecular weight and rheological characteristics. However, to derive such algebraic relationships, more experiments with the materials, each of them with variable molecular weight, would be necessary. Furthermore, specifics concerning molecular weight cannot be determined solely from the data provided by the producers.

The topic of this study was a participation of polymer concentration on the appearance of singularities (beads) along the electrospun nanofibers. It is well known that characterization of polymeric materials differs from batch to batch [48]. It results in a variable value of the concentration separating beaded and beadless nanofibers. The primary factor causing these discrepancies was represented by variable molecular weight modifying rheological characteristics as introduced in Section 2.2. It means that the experimental findings valid for one batch cannot be automatically applied for the other, and the whole evaluation process should be repeated. The rheological procedure presented in the preceding section substantially accelerates acquiring new data (starting concentrations) with new batches, eliminating the necessity to repeat the individual experiments using a spinning device.

## 5. Conclusions

Determination of a starting concentration of electrospinnable polymer solutions for which the obtained electrospun nanofibers exhibit a bead-free surface is usually carried out by a trial-and-error method. The proposed method using oscillatory rheological measurements provided a very good approximation of the starting concentrations. For materials exhibiting first a constant behavior or moderate decrease in a phase angle for lower concentrations, an approximation of the starting concentration is given by a concentration value where a phase angle curve starts to decrease, apparently reflecting a more progressive viscoelastic nature. It was documented using solutions of three different polymeric materials, specifically poly(vinylidene fluoride-*co*-hexafluoropropylene), poly(vinyl butyral), and poly(ethylene oxide).

## Figures and Tables

**Table 1:** Summary of prepared concentrations including molecular weights (*M*_w_) of used polymeric materials.

Material	Molecular Weight (g/mol)	Concentration (%)
PVDF-*co*-HFP (solvent *N*,*N′*-dimethylformamide)	455,000 ^a^	8, 10, 12, 13, 15, 17, 18, 20–29 in steps of 1%
PVB (solvent ethanol)	75,000 ^a^	3–10 in steps of 1%, 12, 14
PEO (solvent distilled water)	637,500 ^b^	0.3, 0.5, 0.6, 0.8, 1, 1.2, 1.5, 1.8, 2—6 in steps of 0.5%, 7, 7.5, 8, 9

^a^ Molecular weight provided by the producer; ^b^ molecular weight determined by SEC (Size Exclusion Chromatography).

**Table 2:** Summary of the individual concentrations for all three used polymeric materials.

Material	Overlap Concentration c* (wt.%)	Entanglement Concentration *c*_e_* (wt.%)	Starting Concentration *c*_start_ (wt.%)
PVDF-*co*-HFP (solvent DMF)	12	19	22
PVB (solvent ethanol)	5	8	7.5
PEO (solvent distilled water)	1.4	4.6	6

## Data Availability

The data presented in this study are available on request from the corresponding author.
